# Women and Cardiovascular Disease: The Gender Gap—A Life-Course and Sex-Specific Perspective

**DOI:** 10.3390/jcdd13070316

**Published:** 2026-07-08

**Authors:** Sandra Eifert

**Affiliations:** University Department of Cardiac Surgery, Leipzig Heart Center, 04289 Leipzig, Germany; sandra.eifert@medizin.uni-leipzig.de

Cardiovascular disease (CVD) remains the leading cause of death among women worldwide and accounts for a substantial proportion of global morbidity, mortality, and disability, as well as healthcare expenditure. Although age-adjusted cardiovascular mortality rates among women have declined over recent decades in many high-income countries due to advances in prevention, diagnosis, and treatment, significant sex- and gender-related disparities persist across the entire spectrum of cardiovascular care [1,2].

Historically, cardiovascular disease has predominantly been perceived as a male condition. Consequently, women have been underrepresented in clinical trials, translational research, and guideline development for decades. This has contributed to important gaps in knowledge regarding sex-specific pathophysiology, clinical presentation, diagnostic accuracy, therapeutic efficacy, and outcomes. As a result, cardiovascular disease in women often remains underrecognized, underdiagnosed, and undertreated, and is insufficiently prevented.

Recent years have witnessed growing awareness of the need to address these inequities. International scientific societies and public health initiatives increasingly emphasize the integration of sex and gender into cardiovascular research and clinical practice. Nevertheless, substantial disparities remain, particularly among women from socioeconomically disadvantaged populations, racial and ethnic minority groups, and regions with limited healthcare access.

A comprehensive understanding of cardiovascular health in women requires a life-course approach. Female cardiovascular risk is shaped not only by traditional risk factors such as hypertension, dyslipidemia, diabetes mellitus, obesity, smoking, and physical inactivity, but also by sex-specific biological factors and gender-related determinants of health. Importantly, hormonal transitions and reproductive milestones represent critical windows of opportunity for cardiovascular risk assessment and prevention.

Particular attention should be given to key transitional phases in a woman’s life, including menarche, pregnancy, the postpartum period, infertility, polycystic ovary syndrome, premature ovarian insufficiency, menopause, and healthy aging. Pregnancy, in particular, functions as a natural cardiovascular stress test. Adverse pregnancy outcomes—including hypertensive disorders of pregnancy, gestational diabetes, preterm delivery, fetal growth restriction, and recurrent pregnancy loss—are increasingly recognized as early markers of future cardiovascular disease and should be incorporated into routine cardiovascular risk stratification [2,3,4,5,6,7].

Beyond hormonal influences, social, behavioral, environmental, and psychosocial factors contribute substantially to cardiovascular health. Gender-related determinants—including healthcare access, caregiving responsibilities, socioeconomic status, health literacy, occupational stress, and implicit bias within healthcare systems—can influence disease development, healthcare utilization, and clinical outcomes. Notably, psychosocial stress, depression, anxiety, trauma exposure, and chronic caregiving burden appear to exert a particularly strong impact on women’s cardiovascular health and are increasingly recognized as important yet often overlooked risk factors.

This evolving field highlights the need to address both traditional and female-specific cardiovascular risk factors. Particular emphasis should be placed on sex-related differences in disease mechanisms, clinical manifestations, and outcomes across a broad spectrum of cardiovascular conditions. These include coronary artery disease, especially ischemia with non-obstructive coronary arteries (INOCA), coronary microvascular dysfunction, spontaneous coronary artery dissection (SCAD), myocardial infarction with non-obstructive coronary arteries (MINOCA), heart failure with preserved ejection fraction (HFpEF), valvular heart disease, cardiomyopathies, arrhythmias, aortic diseases, thromboembolic disorders, and cardio-oncology.

Furthermore, advances in precision medicine, cardiovascular imaging, interventional cardiology, cardiac surgery, digital health technologies, and artificial intelligence offer new opportunities to improve cardiovascular prevention and care for women. Understanding sex-specific differences in pharmacology, device therapy, procedural outcomes, and long-term management is essential for delivering equitable and personalized cardiovascular care.

Finally, future research must continue to investigate the biological and sociocultural mechanisms underlying sex and gender disparities in cardiovascular disease. Increasing female representation in clinical trials, incorporating sex-disaggregated analyses, and integrating gender-sensitive approaches into healthcare delivery are critical steps toward closing existing knowledge gaps and improving outcomes for women globally.

A sex- and gender-sensitive, life-course-oriented approach to cardiovascular medicine has the potential not only to reduce disease burden but also to advance precision prevention, early diagnosis, and individualized treatment strategies, ultimately improving cardiovascular health and longevity for women across all stages of life [8].

In this Special Issue, we assembled eight contributions that collectively highlight key aspects of cardiovascular health across the female life course.

Two articles focus on pregnancy-related cardiovascular diseases, with particular emphasis on peripartum cardiomyopathy, addressing current insights into pathophysiology, diagnosis, management, and long-term outcomes [9].

One article investigates the impact of psychosocial stress and other psychosocial risk factors in women and men following left ventricular assist device (LVAD) implantation. Drawing on data from the INTERMACS Registry, this study provides important insights into sex-related differences in psychological burden and post-implantation outcomes.

Three articles examine sex-specific aspects of ischemic heart disease in women, exploring differences in pathophysiological mechanisms, clinical presentation, disease onset, and diagnostic challenges. Particular attention is given to factors that may contribute to the less favorable recognition and management of ischemic heart disease in women compared with men.

Another contribution explores the potential benefits of gender-tailored Heart Team decision-making and discusses how incorporating sex- and gender-related considerations into multidisciplinary cardiovascular care may improve patient outcomes.

To further advance personalized and sex-sensitive cardiovascular care, one article addresses drug intolerance and individualized treatment strategies in women with coronary artery disease (CAD), highlighting the importance of considering sex-specific responses to pharmacological therapies.

Together, these articles underscore the importance of integrating sex- and gender-specific perspectives into cardiovascular research and clinical practice and contribute to a more comprehensive understanding of cardiovascular disease in women throughout the life course.
